# Evaluation of Xpert MTB/XDR test for susceptibility testing of *Mycobacterium tuberculosis* to first and second-line drugs in Uganda

**DOI:** 10.1371/journal.pone.0284545

**Published:** 2023-08-17

**Authors:** Achilles Katamba, Willy Ssengooba, James Sserubiri, Derrick Semugenze, George William Kasule, Abdunoor Nyombi, Raymond Byaruhanga, Stavia Turyahabwe, Moses L. Joloba

**Affiliations:** 1 Department of Medicine, School of Medicine, Clinical Epidemiology and Biostatistics Unit and Uganda Implementation Research Consortium, Makerere University, Kampala, Uganda; 2 Department of Medical Microbiology, Makerere University, Kampala, Uganda; 3 Lung Institute, Makerere University, Kampala, Uganda; 4 Biomedical Research Center, Makerere University, Kampala, Uganda; 5 Ministry of Health, National Tuberculosis, and Leprosy Programme, Kampala, Uganda; Rutgers Biomedical and Health Sciences, UNITED STATES

## Abstract

**Background:**

Drug-Resistant Tuberculosis (DR-TB) is one of the major challenges to TB control.

**Design and methods:**

This was a blinded, laboratory-based cross-sectional study using sputum samples or culture isolates. Samples were from patients with rifampicin-resistant—TB and/or with high risk for isoniazid (INH) resistance and/or 2^nd^ line fluoroquinolones (FQ) and injectable agents (IAs). The diagnostic accuracy of the Xpert^®^ MTB/XDR test was compared to MGIT960 and the Hain Genotype^®^ MTBDR*plus* and MDRsl assays (LPA) as reference DST methods. Factors for laboratory uptake of the Xpert^®^ MTB/XDR test were also evaluated.

**Results:**

Of the 100 stored sputum samples included in this study, 65/99 (65.6%) were resistant to INH, 5/100 (5.0%) were resistant to FQ and none were resistant to IAs using MGIT960. The sensitivity and specificity, n (%; 95% Confidence Interval, CI) of Xpert^®^ MTB/XDR test for; INH was 58 (89.2; 79.1–95.5) and 30 (88.2; 72.5–96.6) and for FQ; 4 (80.0; 28.3–99.4) and 95 (100; 96.2–100), respectively. Using LPA as a reference standard, a total of 52/98 (53.1%) were resistant to INH, 3/100 (3.0%) to FQ, and none to IA. The sensitivity and specificity, n (%; 95%CI) of Xpert^®^ MTB/XDR test compared to LPA for; INH was 50 (96.1; 86.7–99.5) and 34 (74.0; 58.8–85.7) for FQ 3 (100; 29.2–100) and 96 (99.0; 94.3–99.9) respectively. The factors for laboratory uptake and roll-out of the Xpert^®^ MTB/XDR test included: no training needed for technicians with, and one day for those without, previous Xpert-ultra experience, recording and reporting needs were not different from those of Xpert-ultra, the error rate was 4/100 (4%), one (1%) indeterminate rate and test turn-around-time were 1hr/45 minutes.

**Conclusion:**

There is high sensitivity and specificity of Xpert^®^ MTB/XDR test for isoniazid and fluoroquinolones. There are acceptable Xpert^®^ MTB/XDR test attributes for the test uptake and roll-out.

## Introduction

Tuberculosis (TB), caused by *M*. *tuberculosis* (MTB), remains an important cause of global morbidity and mortality [1]. Moreover, the rates of drug-resistant TB (DR-TB) are increasing in many countries. Globally, the burden of DR-TB is estimated to have increased between 2020 and 2021, with 450 000 (95% UI: 399 000–501 000) new cases of rifampicin-resistant TB (RR -TB) in 2021 [1]. Rapid DR-TB diagnosis followed by timely initiation to appropriate treatment remains a high priority [2]. In 2021, Uganda with an estimated population of 46 million people, had a TB incidence of 199(119–298)/100,000 population and an MDR/RR-TB incidence of 63(38–98)/100,000 population. Only 69% of the TB cases were tested with a rapid diagnostic test and 75% tested for rifampicin resistance (RR) at the time of diagnosis. Only 58% of the laboratory-confirmed MDR/RR-TB were tested for fluoroquinolone (FQ) resistance.

There is still a huge need to increase DR-TB diagnosis and treatment initiation. In Uganda, Drug susceptibility testing (DST) methods for MTB are mainly by the solid agar proportions method and the liquid-based system: Mycobacterial Growth Indicator Tube (MGIT960^®^) system (Becton Dickinson, Sparks, Maryland). The agar proportions method relies on the use of media generally prepared within the laboratory, while the MGIT system can be purchased as a kit. Culture-based DST methods require highly specialized laboratories with advanced biosafety standards and highly skilled personnel. Therefore, a genotypic susceptibility testing methodology with the kit format and automated DNA-based technology that detect resistance-conferring mutations rather than culture-based DST may be advantageous. The Line Probe Assay (LPA) i.e. Genotype^®^ MTBDR*plus* and MDRsl (Hain Life Sciences, Nehren, Germany) provides an alternative rapid, but less accessible molecular MDR-TB and preXDR-TB diagnostic. Although LPA offers DST results to rifampicin, isoniazid (INH), FQs, and 2^nd^ line injectable agents (IAs) in a short time than culture-based DST methods, it requires extra technical skills as well as infrastructure requirements such as the need for three rooms (pre-amplification, amplification, and post-application).

As part of the efforts to reduce these challenges and the diagnostic gap for MDR-TB, the WHO endorsed the use of GeneXpert MTB/RIF test (Xpert; Cepheid, Sunnyvale, CA) in 2011, which was upgraded to GeneXpert Ultra (Ultra) in 2017, as the initial diagnostic test for TB and individuals at high-risk of RR-TB [3]. This was followed by the WHO End TB strategy recommendation for universal screening for DR-TB, TB treatment informed by drug resistance patterns, and the use of shorter regimens with drugs that are more effective [4]. Achieving this recommendation remains a challenge in most of the low and middle-income countries (LMICs) [5, 6]. Most of the LMICs including Uganda are basing MDR-TB treatment initiation on the Xpert results only i.e. patients with RR-TB detected on Xpert are initiated on MDR-TB treatment. Few patients receive 2^nd^ line DST before MDR-TB treatment initiation.

Cepheid developed a novel cartridge, the Xpert^®^ MTB/XDR test (launched in 2021), which is capable of determining susceptibility to INH, FQs, and 2^nd^ line IAs. This test is currently recommended as a reflex test among TB patients with high-risk INH resistance as well as patients with a high risk of resistance to 2^nd^ line anti-TB drugs. In 2021, the WHO endorsed and recommended additional field evaluation of the Xpert^®^ MTB/XDR test before final endorsement. The current study aimed at evaluating the performance of the Xpert^®^ MTB/XDR test for MTB susceptibility testing among presumptive XDR-TB patients using biobanked specimens. The test performance indicators were compared to those of Xpert Ultra test.

## Materials and methods

### Ethical considerations

The study received Research Ethics committee approval from the Makerere University School of Biomedical Sciences Research Ethics Committee (SBS-2021-19) and the Uganda National Council of Science and Technology (HS1395ES). Administrative approval to use anonymized stored samples were obtained from the National TB and Leprosy programme, Ministry of Health Uganda and from the Mycobacteriology (BSL-3) laboratory, Department of Medical Microbiology, Makerere University. The study received waiver of consent since it used fully anonymized stored samples.

### Study design and population

This was a single-blinded, laboratory-based cross-sectional study to determine the performance of the Xpert^®^ MTB/XDR test using stored sputum samples collected between January 2020 and December 2021. Samples were from patients with rifampicin resistance and/or INH resistance and those with high risk of INH-mono-resistance for detection of resistance to INH and/or 2^nd^ line FQs and IAs. Results were compared with those from the WHO endorsed MGIT 960 DST or Hain MDR*plus* and MTBDR*sl* as reference comparator. Expectorated sputum samples included in this study were those stored at -80°C and with results reported as such by the National TB Reference Laboratory (NTRL) or by Mycobacteriology (BSL-3) laboratory at Makerere University. The results of the investigational Xpert^®^ MTB/XDR test were not used for clinical care. Testing with Xpert^®^ MTB/XDR test were conducted between 11/September 2021 to 26/May /2022. All operators performing investigational tests were blinded by the data managers and the investigator to the previous DST results of the sample. The Xpert^®^ MTB/XDR assay was performed by different operators and each operator was blinded to the other investigational test’s result. To be eligible, the stored sample had to have a corresponding MTB isolate for repeat phenotypic and genotypic DST if needed. However, efforts were made to obtain the reference standard DST results from tests previously done from the two reference laboratories. Stored sputum samples were selected by convenient sampling from previously treated TB patients at high risk of mono isoniazid resistance and/or from patients diagnosed with rifampicin resistance who are at high risk of having resistance to FQs and/or IAs.

### Laboratory procedures

All study procedures were performed according to written Standard Operating Procedures (SOPs). Well-selected stored sputum samples were checked for the availability of reference test results. Retrieved samples were processed according to the SOPs of the laboratory for the reference standard tests (MGIT960 DST and/or Hain MDR*plus* and MTBDR*sl*), in case the results were missing from the testing laboratory where the sample is obtained or required repeat testing, as well as according to the manufacturer’s instructions for the Xpert^®^ MTB/XDR test.

Briefly, The Xpert^®^ MTB/XDR test detects susceptibility to INH, ethionamide, FQs, and IAs (amikacin, kanamycin, and capreomycin), the key drugs used in the management of MDR-TB. It is a semi-quantitative nested PCR followed by high-resolution technology running on the gene Xpert platform. The targets per drug include; INH gene target, *inhA* promotor with -1 to -32 intergenic nucleotide, *katG* codon 311–319, nucleotide 939–957, *fabG1* codon 199–210, nucleotide 597–630, *oxyR-ahpC* with nucleotide -5 to 50 or intergenic -47 to 92. For ethionamide, the gene target is *inhA* promoter with nucleotide -1 to -32 intergenic. For FQs, the gene targets are *gyrA* at codons 87–95 nucleotides 261–285 and *gyrB* at codons 53–544 or 493–505 with nucleotide 1596–1632 [7]. The targeted genes for 2^nd^ line IAs include; amikacin and kanamycin, gene target *rrs* with nucleotide 1396–1417 and capreomycin with gene target *eis* promoter, nucleotides -6 to -42 intergenic [8].

### Statistical analysis

Repeat testing was done in case of discordant results defined as; a) Samples with any resistance or indeterminate on the Xpert^®^ MTB/XDR test but no resistance detected by any of the reference standard tests, b) Samples which have no resistance detected on Xpert^®^ MTB/XDR test but with resistance to isoniazid and/or 2^nd^ line drugs on any of the reference standard tests. For the analysis of the diagnostic accuracy analysis, the Xpert^®^ MTB/XDR test was performed on stored sputum or specimens meeting criteria for indeterminate cases after two repeats were excluded. The main analyses comparing Xpert^®^ MTB/XDR test to MGIT-960 DST and Hain MDR*plus* and MTBDR*sl* (reference standard tests) results consisted of constructing exact 95% confidence intervals (95% Cis) around the proportions of interest: sensitivity, specificity, prediction positive and negative values. For feasibility analysis, descriptive statistics were used to calculate and compare test error rates, the number of hours required for training, training runs required, the time required to run each test, and test indeterminate rates.

## Results

A total of 100 stored sputum samples were included in this study of which, 80 (80%) were resistant to rifampicin, 9 (9.0%) were isoniazid mono-resistant, and 11(11.0%) were susceptible to isoniazid and rifampicin obtained from previously treated TB patients with high-risk resistance. Of the samples tested using MGIT960 phenotypic DST, 65/99 (65.6%) were resistant to isoniazid (INH), 5/100 (5.0%) resistant to fluoroquinolones (FQ) and none was resistant to the injectable agent (IA). The sensitivity and specificity, n (%; 95%Confidence Interval, CI) of the Xpert^®^ MTB/XDR test for; INH were 58 (89.2; 79.1–95.5) and 30 (88.2; 72.5–96.6), FQ 4 (80.0; 28.3–99.4) and 95 (100; 96.2–100), respectively. The specificity for IAs was 100 (100; 96.3–100), Table 1.

**Table 1:** Diagnostic accuracy of Xpert^®^ MTB/XDR test using phenotypic drug susceptibility test as a reference comparator.

Drug (n)		R	S	Sensitivity n(%; 95% CI)	Specificity n(%; 95%CI)	PPV % (95%CI)	NPV % (95%CI)
**INH (99)**	**R**	58	4	58 (**89.2**; 79.1–95.5)	30 (**88.2**; 72.5–96.6)	**93.5** (84.2–98.2)	**81.1** (64.8–92.0)
	**S**	7	30				
**FQ (100)**	**R**	4	0	4 (**80.0**; 28.3–99.4)	95 (**100**; 96.2–100)	**100** (39.7–100)	**99.0** (94.3–99.9)
	**S**	1	95				
**IAs (100)**	**R**	—	—	N/A	100 (**100**; 96.3–100)	N/A	**100** (96.3–100)
	**S**	—	100				

Key: INH = Isoniazid, FQ = Fluoroquinolone, IAs = Injectable agents, N = Number, R = Resistant, S = Sensitive, CI = Confidence Interval, PPV = Positive Predictive Value, NPV = Negative Predictive Value, N/A = Not Applicable, * = Xpert^®^ MTB/XDR test indeterminate (n = 1).

Using LPA as a reference standard, a total of 52/98 (53.1%) were resistant to INH, 3/100 (3.0%) to FQ, and none to IA. The sensitivity and specificity, n (%; 95%CI) of Xpert^®^ MTB/XDR test compared to LPA for; INH was 50 (96.1; 86.7–99.5) and 34 (74.0; 58.8–85.7) and FQ 3 (100; 29.2–100) and 96 (99.0; 94.3–99.9) respectively. All samples were sensitive to IAs leading to, 96 (100; 96.2–100) specificity for IAs, Table 2.

**Table 2:** Diagnostic accuracy of Xpert^®^ MTB/XDR test using hain MDR*plus* and MTBDR*sl* as a reference comparator.

Drug (n)		R	S	Sensitivity n(%; 95%CI)	Specificity n(%; 95%CI)	PPV % (95%CI)	NPV % (95%CI)
**INH** (98*)	**R**	50	12	50 (**96.1**; 86.7–99.5)	34 (**74.0**; 58.8–85.7)	**80.6** (68.6–89.5)	**94.4** (81.3–99.3)
	**S**	2	34				
**FQ (100)**	**R**	3	1	3 (**100**; 29.2–100)	96 (**99.0**; 94.3–99.9)	**75** (19.4–99.3)	**100** (96.2–100)
	**S**	0	96				
**IAs (98)**	**R**	—	—	N/A	96 (**100**; 96.2–100)	N/A	**98.0** (92.8–99.7)
	**S**	1	96				

Key: INH = Isoniazid, FQ = Fluoroquinolone, IAs = Injectable Agents, N = Number, R = Resistant, S = Sensitive, CI = Confidence Interval, PPV = Positive Predictive Value, NPV = Negative Predictive Value, N/A = Not Applicable. * = LPA inconclusive (n = 1) and Xpert^®^ MTB/XDR test indeterminate (n = 1) on different samples.

The factors for laboratory uptake of the Xpert^®^ MTB/XDR test were mainly: no training needed for technicians with previous Xpert ultra experience and 1 day for those without, recording and reporting needs were not different from those of Xpert ultra. The error rate was 4/100 (4%), only one (1%) result was indeterminate/uninterpretable, test turn-around-time was 1:45 minutes and workflow was similar to that of Xpert ultra.

## Discussion

Results from this in-country evaluation study show that the Xpert^®^ MTB/XDR test has high sensitivity and specificity regarding isoniazid (INH) and fluoroquinolone (FQs) compared to phenotypic and line probe assay (LPA) Drug Susceptibility Testing (DST). Better diagnostic accuracy measures were recorded when LPA was used as a reference standard. The test achieved acceptable attributes in terms of training needs, test Turn-Around Time (TAT), indeterminate and error rates as well as test workflow. The high sensitivity for both INH and FQs obtained in this study is in agreement with the previous evaluation studies including a Cochrane review [7, 9, 10]. The lower specificity of the Xpert^®^ MTB/XDR test in our study may be attributed to the study design which used the previously recorded results of the reference standard, and in some cases, *M*. *tuberculosis* isolates were unavailable to repeat the reference test.

The Xpert MTB/XDR test attributes were comparable to those of Xpert Ultra with DST results generated in less than 90 minutes with limited technical and infrastructural requirements. The test exhibits better technical and operational attributes compared to other WHO-endorsed molecular diagnostics for DR-TB as previously noted [11, 12]. These performance attributes makes the Xpert MTB/XDR test ideal for reducing the current challenges of obtaining INH and FQs DST results for better DR-TB patient management.

The WHO recommends that in people with bacteriologically confirmed pulmonary TB, low complexity automated Nucleic Acid Amplification Tests (NAATs) may be used on sputum for the initial detection of resistance to INH and FQ, rather than culture-based phenotypic DST. Early diagnosis and DST forms a stronger pillar for DR-TB management and control [4]. Molecular rapid diagnosis/DST followed by rapid appropriate treatment initiation provides greater benefits for patient management as well as DR-TB control. Therefore, a TB diagnostic algorithm including Xpert MTB/XDR test will reduce the diagnostic challenges needed for tests that are requested from the reference laboratories such as LPA and culture-based DST [2].

In most of the LMICs, TB patients are initiated on treatment with limited or no INH and/or 2^nd^ line DST results. If results of FQ DST are not available at the initiation of DR-TB treatment, it may increase the risk of acquired bedaquiline resistance in undetected FQ resistance. This is even more important in the implementation of the newly recommended second-line bedaquiline containing regimen [13]. In some settings over 30% of the RR-TB patients are already resistant to FQ, i.e. pre-XDR, at the start of the treatment [14]. Therefore, implementing the Xpert MTB/XDR test means that more patients will be initiated to DST-guided TB treatment regimen. This is likely to lead to better MDR-TB treatment outcomes as well as better estimates of resistance to INH and FQs the two key drugs in the current standard regimens for the management of DR-TB [2]. However, owing to the recent change in the definition, the present version of the Xpert MTB/XDR test is not fully capable of detecting WHO-defined XDR-TB, but pre-XDR-TB. This makes complementary use of alternative DST methods including phenotypic and genotypic methods of high importance for providing universal DST in countries with a high prevalence of DR-TB [15].

Our study had some limitations: These include, the low number of samples with resistance to FQs and none resistant to injectable agents due to the fact that Uganda is a low MDR/RR-TB setting. This may have lowered our confidence in the true estimates of sensitivity for these drugs, but it increases confidence in specificity and makes it a suitable setting where Xpert MTB/XDR test is most needed.

We used previously stored sputum and/or isolates which may have reduced the sensitivity, however, the direct testing was done on molecular tests and, in case of need for repeat indirect testing was done on available cultured isolates. Isolates of three of the seven with discordant results between phenotypic and Xpert MTB/XDR test, that required repeat testing failed to grow. The seven phenotypic DST INH resistant but Xpert MTB/XDR test INH sensitive, only one was INH resistant using LPA. Nonetheless, genome sequencing of these isolates would be helpful to crosscheck mutations on other gene targets that could cause INH resistance. Similar discordance between phenotypic and genotypic DST have been previously documented elsewhere [16, 17].

The repeated discordant results had mutations *katG* (n = 2) and *fabG1* (n = 2) on Xpert MTB/XDR test and results did not change on repeat, S1-S4 Tables in S1 File. Some of the data was missing and we could not relate the accuracy to patient’s clinical and demographic data. Furthermore, the evaluation of the Xpert MTB/XDR test using a third composite reference standard such as clinical outcome as well as response to treatment in addition to phenotypic and LPA DST, would have more impact.

Our study did not test for ethionamide, a drug also included in the Xpert MTB/XDR test and in the management of MDR/RR-TB, because this is not tested phenotypically in Uganda, however, a recent study found DST for ethionamide to have suboptimal sensitivity on Xpert MTB/XDR test due to inclusion of only mutations in the *inhA* promoter region [18]. In our study, all samples tested showed no resistance to ethionamide since no *inhA* mutations were detected using Xpert MTB/XDR test. Future evaluation studies in settings where ethionamide is tested phenotypically are recommended.

## Conclusion

There is high sensitivity and specificity of Xpert^®^ MTB/XDR test for isoniazid and fluoroquinolones. There are acceptable Xpert^®^ MTB/XDR test attributes for the uptake and roll-out. We recommend the initial roll-out phase to consider prioritization of all DR-TB treatment health facilities to have Xpert MTB/XDR testing machines which can run both Xpert Ultra and XDR tests.

For better uptake and roll-out, countries should consider that all new GeneXpert machine procurements are Xpert MTB/XDR for easy integration. In the future, the manufacturer should plan to swap Xpert Ultra with Xpert MTB/XDR modules to ensure better access to XDR test in all GeneXpert sites.

## Supporting information

S1 File Evaluation of Xpert MTB/XDR test in Uganda. (XLS)
